# The Impact of COVID-19 Infection Control Measures on End-Stage Renal Disease Patients in a Community Hemodialysis Setting

**DOI:** 10.7759/cureus.43114

**Published:** 2023-08-08

**Authors:** Behram A Khan, Rajat Tagore, Shilpa Rastogi, Yan Hua, Vincent W See, XiaoJie Qu, Hwee Lin Wee, Celestine Grace X Cai

**Affiliations:** 1 Medicine, National University of Singapore, Singapore, SGP; 2 Renal Medicine, Ng Teng Fong General Hospital, Singapore, SGP; 3 Medical Affairs, The National Kidney Foundation Singapore, Singapore, SGP; 4 Public Health, National University of Singapore, Singapore, SGP

**Keywords:** end stage renal disease (esrd), infection control measures, dialysis center, community-based setting, sars-cov-2, health services disruption, pandemic preparedness, chronic hemodialysis, hd (hemodialysis), covid 19

## Abstract

Introduction: Several non-pharmaceutical infection control measures have been implemented at community-based hemodialysis centers to reduce the risk of Coronavirus Disease of 2019 (COVID-19) transmission, caused by the SARS-CoV-2 virus. However, there have been concerns that such measures may disrupt the routine and timely care required by patients, with adverse effects on their health outcomes. This cross-sectional study aims to determine the unintended consequences of COVID-19 infection control measures on hemodialysis patients.

Methods: Electronic medical records were extracted from patients enrolled in community-based hemodialysis centers in Singapore. A baseline group prior of patients consisted of those enrolled in 2017, which was three years prior to the SARS-CoV-2-related pandemic (n = 548). This was compared with the study group of patients enrolled in 2019 (n = 426), just before the COVID-19 pandemic started. Medical records for these two groups were extracted from January to July 2018 for the baseline group and from January to July 2020, respectively. Three regression models were built to study dialysis adherence, kidney disease biomarkers, and hospitalization episodes.

Results: There was no statistically significant difference in hospitalization and mortality outcomes, adherence to dialysis management, laboratory results for dialysis-related clearance, and anemia outcomes. There was a higher proportion of patients hospitalized for vascular access-related reasons in the study group as compared to the baseline group (OR 1.6, 95% CI: 1.10 to 2.29, P = 0.014). Patients in the study group had albumin levels 2.13% higher (95% CI: 0.88 to 3.39, P < 0.001) and alkaline phosphatase levels 7.3% lower (95% CI: 1.17 to 13.02, P = 0.020) than those in the baseline group.

Conclusions: From this community-based hemodialysis study in Singapore, it was shown that the COVID-19 pandemic did not disrupt regular healthcare services for these patients. With strategies instituted for a coordinated health delivery workflow, ensuring sufficient capacity in the various healthcare delivery sites and overall pandemic preparedness, the patient clinical outcomes measures continued to be met with no adverse consequences noted. Some improvements in dialysis-related laboratory values and quality of care targets may be due to more stringent measures instituted to protect these vulnerable patients in the community.

## Introduction

Patients with end-stage renal disease (ESRD) are considered immunocompromised and are vulnerable to higher morbidity or mortality with SARS-CoV-2 infection during the coronavirus disease of 2019 (COVID-19) pandemic [1,2]. Hemodialysis (HD) centers have such patients interacting with their care team of medical staff, in a unique environment regarding prolonged and repeated contact. Such exposure is usually four hours for each HD session, which occurs usually three times a week. This leads to healthcare sites that pose a substantial risk of SARS-CoV-2 transmission and related COVID-19 infection [3].

To contain the spread of SARS-CoV-2 infection, the Singapore government raised the Disease Outbreak Response System Condition (DORSCON) status from yellow to orange, with enhanced COVID-19 infection control measures being implemented. These included quarantine for suspected cases, temperature screening in public spaces, visitor restrictions in hospitals, and a two-month social isolation or “lock down” period [4,5].

In community-based hemodialysis centers, temperature screening was conducted at dedicated entrance points. Patients with a temperature higher than 37.5 degrees Celsius were redirected to a COVID-19 response clinic for further testing. Facial masks were required within dialysis centers for everyone, with preference given to surgical masks [6]. Patients and their care staff were assigned to dedicated shifts, with separation of each shift being instituted to minimize intermingling. Telemedicine resources were enabled to replace in-person patient assessments by nephrologists [7]. This was to reduce the cross-institution spread of infection, as these nephrologists were primarily deployed for their duties at the hospitals. Kidney transplants for patients that were on the waiting list were deferred, if clinically appropriate [8]. The latter was done to reserve hospital capacity to deal with emergencies and defer elective surgical cases.

Although the above measures were meant to reduce the spread of infection in a vulnerable population, it was felt that there would be unintended consequences. There was evidence of such incidences with prior experiences of caring for HD patients in pandemic environments. During the severe acute respiratory syndrome (SARS) outbreak in 2003, hospitals in Taiwan mandated the use of N95 masks during HD sessions but this resulted in patients having reduced blood oxygen saturation and increased occurrence of adverse respiratory events [9]. Nephrologists noted challenges of performing telemedicine consultations, including the inability to perform proper physical examinations to assess the patients and the reliance on the subjective assessments performed by on-site healthcare staff at the dialysis centers [7]. Furthermore, the deferment of elective surgeries and interventional procedures, such as postponement of HD vascular access procedures and kidney transplants was thought to increase mortality and lead to poor transplant outcomes [10,11].

With Singapore’s experience with the SARS outbreak, the health authorities were quick to recognize the potential gravity of the situation and implemented strict preventive measures early in the COVID-19 pandemic. This study was conducted to understand and assess the unintended consequences of COVID-19 control measures on ESRD patients receiving HD in community-based dialysis centers. The results would lead to better planning and institution of patient-centric prevention measures, for similar infectious disease pandemics in the future.

## Materials and methods

Study design and subjects

National University of Singapore Institutional Review Board issued approval NUS-IRB-2020-464, with waiver of consent for using patients’ clinical audit data.

The study population consisted of patients older than 21 years of age, undergoing HD at community-based dialysis centers in Singapore. This cross-sectional study compares outcomes of HD patients undergoing their routine chronic dialysis treatments during the infection control measures instituted during the COVID-19 pandemic, with historic controls in the same dialysis centers prior to the pandemic and these measures. The implementation of DORSCON Orange status by the regulatory authorities on February 7, 2020 was used as the timeline for the implementation of enhanced infection control measures [12]. Patients who had joined the dialysis centers from January 1, 2019 to December 31, 2019 were placed in the study group and their data was extracted for analysis from January 1, 2020 to August 6, 2020. Patients who joined the dialysis centers from January 1, 2017 to December 31, 2017 were classified as the historical control group and their data were extracted for analysis from January 1, 2018 to August 6, 2018.

Data collection

Demographics and comorbidity information was routinely entered into the electronic medical record (EMR), at the time of entry in the HD program. Medication and hospitalization data was also entered in the EMR by the nurses after every change in the patient's treatment regimen and after each hospitalization. Mortality data was compiled by the Medical Affairs department when the patient died. All this data was compiled in a de-identified and aggregate format by performing a retrospective EMR audit by the data team in the Medical Affairs department and passed to the study team for review. The study team then analyzed these data sets and any requests for additional information or clarification were forwarded to the Medical Affairs data team, as and when needed.

Kt/V values were used as a marker to measure dialysis adequacy, and this was recorded after each dialysis session as part of the routine HD patient care. Laboratory indicators of bone mineral disease, anemia and nutrition were monitored [13,14]. These included intact parathyroid hormone (iPTH) levels, calcium level (Ca), phosphate level (PO4), hemoglobin level (Hb), alkaline phosphatase (ALP) and albumin levels, which were recorded as part of routine blood tests. If several such readings were available, the latest measurement from the period under review was used in the analysis. These study parameters were selected to allow comparison and alignment with what was being reported on a national level under the Singapore Renal Registry Annual Report 2021 [15].

Statistical analyses

Characteristics of patients in the study group and the historical control group were compared. Analysis included baseline demographics, hospitalization outcomes and mortality outcomes, as well as blood test results and medication usage. Categorical variables were analyzed using the Fisher’s exact test. Continuous variables were evaluated for normality using the Shapiro-Wilk test. Normally distributed variables were analyzed using t-test and reported as mean with standard deviation. Non-normally distributed variables were analyzed using the Wilcoxon Rank Sum test and reported as median with interquartile range.

Three models were used to analyze the outcome measures obtained from each hospitalization and the values from the routine blood tests. Model 1 shows the crude association between COVID-19 exposure risk and the outcomes of interest. Model 2 looks at the same association but is adjusted for age, while model 3 additionally adjusted for the use of iron supplements to reduce any bias seen in Hb variations.

Logistic regression analysis was conducted for reason of hospitalization, mortality, dialysis adequacy and the blood test result under study. The effect sizes were reported as odds ratios (OR). Mean length of hospitalization and blood test results were transformed to logarithmic scale and linear regression was conducted. The effect sizes were reported as percentage change (% change). The total number of hospitalizations and hospitalizations per patient (for those with at least one hospitalization) were analyzed using either negative binomial regression or zero-truncated negative binomial regression. Their effect sizes were reported as incidence rate ratios (IRR). A p-value of less than 0.05 was considered statistically significant.

## Results

Characteristics of participants

Patients included in the study had a higher proportion of diabetes (75.05%) and cardiovascular disease (60.37%) compared to all patients who were receiving HD at the dialysis centers (diabetes: 65.18%, cardiovascular disease: 50.91%, P < 0.001 for both). Patients included in the study also had higher body mass index (BMI), shorter dialysis vintage, longer dialysis sessions, and a higher total number of medications prescribed (see Table 1).

**Table 1:** Characteristics of patients included in the study, compared to all patients who received hemodialysis in the same dialysis centers IQR: Interquartile range, N: Count

Characteristic	Patients in the study	All patients in the same dialysis centres	P-value
Pre-COVID, N (%)	548 (56.26)	600 (54.55)	-
Peri-COVID, N (%)	426 (43.74)	500 (45.45)	-
Age in years, median (IQR)	64.00 (55.00, 70.00)	64.00 (56.00, 71.00)	0.360
Gender, N (%)			0.873
Male	426 (43.74)	486 (44.18)	
Female	548 (56.26)	614 (55.82)	
Ethnicity, N (%)			0.507
Chinese	546 (56.06)	651 (59.18)	
Indian	84 (8.62)	84 (7.64)	
Malay	312 (32.03)	328 (29.82)	
Other	32 (3.29)	37 (3.36)	
Nationality, N (%)			0.314
Singaporean	945 (97.02)	1076 (97.82)	
Singapore Permanent Resident	29 (2.98)	24 (2.18)	
Comorbid conditions, N (%)			
Diabetes Mellitus	731 (75.05)	717 (65.18)	<0.001
Hypertension	936 (96.10)	1044 (94.91)	0.233
Cardiovascular Disease	588 (60.37)	560 (50.91)	<0.001
Perivascular Disease	218 (22.38)	220 (20.00)	0.203
Body Mass Index, Median (IQR)	24.60 (21.50, 28.20)	24.20 (21.10, 27.40)	0.020
Dialysis vintage in months, Median (IQR)	7.0 (2.0, 10.0)	53.0 (29.0, 105.0)	<0.001
Dialysis duration in minutes, Median (IQR)	255.0 (240.0, 255.0)	240.0 (240.0, 255.0)	0.002
Total number of medications prescribed, Median (IQR)	4.0 (4.0, 5.0)	4.0 (4.0, 5.0)	0.009
Medication prescribed, N (%)			
Erythropoietin	845 (94.20)	797 (87.20)	<0.001
Iron	684 (76.25)	652 (71.33)	0.020
Vitamin D	580 (64.66)	656 (71.77)	0.001
Phosphate binders	827 (92.20)	828 (90.59)	0.257
Calcium channel blockers	349 (38.91)	321 (35.12)	0.105
ACE inhibitors	94 (10.48)	94 (10.28)	0.953
Beta blockers	586 (65.33)	550 (60.18)	0.026
Other anti-hypertensives	69 (7.69)	71 (7.77)	1.000

xOf the 974 patients included in the study, 548 patients were in the historic control group prior to the COVID-19 pandemic (pre-COVID-19) and 426 patients from the study group during the COVID-19 pandemic (peri-COVID-19). In the complete case analysis, which included analyzing their specific blood tests and outcome measures, there were 498 and 399 patients in the pre-COVID-19 and peri-COVID-19 groups, respectively. The reduced numbers were due to the unavailability of some data which was collected as a retrospective clinical audit. The median age was 64 (interquartile range (IQR): 56, 70) in the pre-COVID-19 group and 63 (IQR: 54, 71) in the peri-COVID-19 group (P = 0.108). Dialysis vintage (seven months) and duration on dialysis (255 minutes) for both groups were also similar (see Table 2). Gender, ethnicity, nationality, diabetes mellitus status, hypertension status, cardiovascular disease status, perivascular disease status, BMI, and total number of medications prescribed were not statistically significant between the two groups (see Table 2). There were 78.9% of pre-COVID-19 patients who were prescribed iron medication compared to only 72.9% in the peri-COVID-19 group (P = 0.044).

**Table 2:** Characteristics of patients with (peri-COVID-19) and without (pre-COVID-19) exposure to COVID-19 infection control measures IQR: Interquartile range.  N: Count

Characteristic	Peri-COVID-19	Pre-COVID-19	P-value
Age in years, median (IQR)	64.00 (56.00, 70.00)	63.00 (54.00, 70.75)	0.204
Gender, N (%)			0.713
Male	305 (55.66)	243 (57.04)	
Female	243 (44.34)	183 (42.96)	
Ethnicity, N (%)			0.884
Chinese	311 (56.75)	235 (55.16)	
Indian	47 (8.58)	37 (8.69)	
Malay	174 (31.75)	138 (32.39)	
Other	16 (2.92)	16 (3.76)	
Nationality, N (%)			0.490
Singaporean	534 (97.45)	411 (96.48)	
Singapore Permanent Resident	14 (2.55)	15 (3.52)	
Comorbid conditions, N (%)			
Diabetes Mellitus	408 (74.45)	323 (75.82)	0.678
Hypertension	527 (96.17)	409 (96.01)	1.000
Cardiovascular Disease	331 (60.40)	257 (60.32)	1.000
Peripheral Vascular Disease	114 (20.80)	104 (24.41)	0.206
Body Mass Index, Median (IQR)	24.40 (21.60, 27.82)	24.70 (21.40, 28.77)	0.271
Dialysis vintage in months, Median (IQR)	7 (2, 10)	7 (3, 10)	0.108
Dialysis duration in minutes, Median (IQR)	255 (240, 255)	255 (240, 255)	0.218
Total number of medications prescribed, Median (IQR)	4 (4, 5)	5 (4, 5)	0.675
Medication prescribed, N (%)			
Erythropoietin	472 (94.78)	373 (93.48)	0.496
Iron	393 (78.92)	291 (72.93)	0.044
Vitamin D	318 (63.86)	262 (65.66)	0.622
Phosphate binders	459 (92.17)	368 (92.23)	1.000
Calcium channel blockers	187 (37.55)	162 (40.60)	0.388
ACE inhibitors	56 (11.24)	38 (9.52)	0.467
Beta blockers	322 (64.66)	264 (66.17)	0.689
Other anti-hypertensives	41 (8.23)	28 (7.02)	0.580

Hospitalization and mortality outcomes

There were 739 hospitalizations in the pre-COVID-19 group and 523 hospitalizations in the peri-COVID-19 group (see Table 3). The total number of hospitalizations and number of hospitalizations per patientwase not significantly different between the groups (see Table 4). The peri-COVID-19 group had a 13.5% shorter mean length of stay (95% confidence interval (CI): 0.84% to 24.51%) compared to the pre-COVID-19 group. However, the statistical significance was attenuated after adjusting for age and iron supplementation.

**Table 3:** Outcomes among patients with (peri-COVID-19) and without (pre-COVID-19) exposure to COVID-19 infection control measures GI: gastrointestinal, IQR: Interquartile range, iPTH: Intact parathyroid hormone, N: Count

	Pre-COVID-19	Peri-COVID-19
Hospitalization		
Number of hospitalizations	739	523
Total number of hospitalizations per patient for patients with at least one hospitalization, Median (IQR)	2.00 (1.00, 3.00)	2.00 (1.00, 3.00)
Mean length of stay for patients with at least one hospitalization, Median (IQR)	5.00 (3.00, 8.50)	4.29 (3.00, 8.00)
Top 5 reasons of hospitalization, N (%)		
Vascular Access-Related	244 (33.02)	193 (36.90)
Cardiac Diseases	90 (12.18)	60 (11.47)
Infections	86 (11.64)	58 (11.09)
GI/Liver Disease	48 (6.50)	37 (7.07)
Respiratory Disease	49 (6.63)	26 (4.97)
Mortality, N (%)	29 (5.29)	22 (5.16)
Dialysis adequacy		
Kt/V (>= 1.2%), N (%) within target	494 (99.20)	396 (99.25)
Creatinine-Reduction Ratio, Median (IQR)	67.42 (64.39, 70.21)	68.07 (64.44, 70.56)
Blood tests		
iPTH (16.3 to 33 pmol/L), N (%) within target	113 (22.69)	97 (24.31)
Corrected calcium (8.4 to 9.5 mg/dL), N (%) within target	283 (56.83)	228 (57.14)
Phosphate (3.5 to 5.5 mg/dL), N (%) within target	284 (57.03)	227 (56.89)
Hemoglobin (>= 10g/dL), N (%) within target	421 (84.54)	328 (82.21)
Alkaline phosphatase, Median (IQR)	104.00 (79.00, 147.00)	96.00 (74.00, 132.50)
Albumin, Median (IQR)	39.00 (37.00, 41.00)	40.00 (38.00, 42.00)

The top five causes for hospitalization were due to issues with vascular access, cardiac diseases, infections, gastrointestinal causes, liver dysfunction, and respiratory diseases. Only vascular-access related hospitalizations were significantly different (P = 0.014), with the odds for a vascular-access-related hospitalization in the peri-COVID-19 group being 1.6 times higher (95% CI: 1.10 to 2.29) than that of the pre-COVID-19 group, after adjusting for age and iron supplementation. 

The mortality was 5.29% in the pre-COVID-19 group and 5.16% in the peri-COVID-19 group. The odds of death were not significantly different between the two groups (Model 3, OR: 1.40, 95% CI: 0.58 to 3.40, P = 0.452) of patients in this study (please see Table 4 below).

**Table 4:** Association between exposure to COVID-19 infection control measures and hospitalization or dialysis outcomes CI: Confidence interval, IRR: Incidence rate ratio, OR: Odds ratio, iPTH: Intact parathyroid hormone. Model 1: Unadjusted, Model 2: Adjusted for age, Model 3: Adjusted for age and use of iron medication.

	Model 1		Model 2		Model 3	
	Effect size (95% CI)	P-value	Effect size (95% CI)	P-value	Effect size (95% CI)	P-value
Hospitalization						
Number of hospitalizations, IRR	0.91 (0.77, 1.08)	0.284	0.91 (0.76, 1.08)	0.270	0.95 (0.79, 1.14)	0.585
Total number of hospitalizations per patient for patients with at least one hospitalization, IRR	1.07 (0.81, 1.38)	0.607	1.05 (0.79, 1.37)	0.673	1.12 (0.86, 1.45)	0.366
Mean length of stay for patients with at least one hospitalization, % change	-13.48 (-24.51, -0.84)	0.037	-11.89 (-23.04, 0.87)	0.066	-9.04 (-20.48, 4.05)	0.167
Top 5 reasons of hospitalization, OR (binary)						
Vascular Access-Related	1.42 (1.01, 2.00)	0.046	1.44 (1.02, 2.03)	0.039	1.59 (1.10, 2.29)	0.014
Cardiac Diseases	1.08 (0.71, 1.64)	0.710	1.09 (0.71, 1.65)	0.702	1.03 (0.65, 1.62)	0.902
Infections	0.90 (0.59, 1.36)	0.628	0.90 (0.59, 1.36)	0.626	0.96 (0.61, 1.50)	0.847
Gastrointestinal and Liver Diseases	1.13 (0.67, 1.90)	0.631	1.15 (0.68, 1.92)	0.599	1.14 (0.65, 2.00)	0.642
Respiratory Diseases	0.76 (0.44, 1.29)	0.313	0.75 (0.43, 1.27)	0.292	0.95 (0.53, 1.66)	0.859
Mortality, OR	0.97 (0.55, 1.72)	0.929	1.00 (0.56, 1.77)	0.988	1.40 (0.58, 3.40)	0.452
Dialysis Adequacy						
Kt/V (≥ 1.2%), OR	1.07 (0.23, 5.45)	0.931	1.14 (0.25, 5.85)	0.865	1.12 (0.24, 5.75)	0.886
Creatinine-Reduction Ratio, % change	0.32 (-0.65, 1.29)	0.521	0.57 (-0.37, 1.51)	0.235	0.48 (-0.46, 1.42)	0.319
Blood tests						
Intact parathyroid hormone (16.3 to 33 pmol/L), OR	1.09 (0.80, 1.49)	0.569	1.14 (0.83, 1.56)	0.414	1.15 (0.84, 1.57)	0.396
Corrected calcium (8.4 to 9.5 mg/dL), OR	1.01 (0.78, 1.32)	0.924	1.01 (0.78, 1.32)	0.921	1.01 (0.77, 1.31)	0.966
Phosphate (3.5 to 5.5 mg/dL), OR	0.99 (0.76, 1.30)	0.967	1.01 (0.78, 1.32)	0.919	1.02 (0.78, 1.33)	0.891
Hemoglobin (≥ 10g/dL), OR	0.84 (0.59, 1.20)	0.350	0.85 (0.60, 1.22)	0.379	0.85 (0.60, 1.21)	0.372
Alkaline phosphatase, % change	-7.38 (-13.08, -1.31)	0.018	-7.43 (-13.14, -1.35)	0.018	-7.28 (-13.02, -1.17)	0.020
Albumin, % change	2.39 (1.13, 3.67)	<0.001	2.18 (0.93, 3.44)	<0.001	2.13 (0.88, 3.39)	<0.001

Dialysis adequacy and blood tests

Comparison of dialysis adequacy and dialysis outcomes related to blood tests results were similar in the pre-COVID-19 and peri-COVID-19 groups for Kt/V (≥ 1.2%), iPTH (16.3 to 33 pmol/L), corrected Ca (8.4 to 9.5 mg/dL), PO4 (3.5 to 5.5 mg/dL) and Hb (≥ 10 g/dL). ALP levels were 7.28% (95% CI: 1.17 to 13.02%) lower in the peri-COVID-19 group compared to the pre-COVID-19 group (P = 0.020). The peri-COVID-19 group had albumin levels 2.13% higher (95% CI: 0.88 to 3.39%) than that of the pre-COVID-19 group (P ≤ 0.001).

## Discussion

Characteristics of patients

Both the pre-COVID-19 patients and peri-COVID-19 patient groups had similar characteristics, making the pre-COVID-19 group a valid historical control. Iron supplementation was significantly different between the two groups, but this is accounted for through statistical adjustment during subsequent analysis.

The patients included in this study were those who joined the HD program at the dialysis centers within one year prior to the start of their respective study periods. It is expected that their characteristics may be different from those who were already enrolled and undergoing their chronic dialysis at these centers for a variable amount of time. Most notably, these newly enrolled patients had more complex comorbidities compared to existing patients, as they may be suffering from acute complications resulting in the necessity to initiate them on chronic dialysis. It was observed that they had more medications prescribed, higher BMI including fluid weight, and longer dialysis durations required to remove excess water.

Hospitalization and mortality

Hospitalization rates were not significantly affected by infection control measures (IRR: 0.95, 95% CI: 0.79 to 1.14, P = 0.585), as detailed in the results above (Table 4). The analysis suggests that there was a lower hospitalization rate in the peri-COVID-19 group compared to the pre-COVID-19 group. A study comparing ESRD patients' outcomes prior to and after the introduction of telemedicine for nephrologist assessment during the COVID-19 pandemic, found comparable results where telemedicine resulted in lower hospitalization rates [16]. The lack of statistical significance in our study may be attributed to the smaller sample size and the difference in the study population since the study referenced included all HD patients instead of only those who had recently joined the dialysis centers [16].

The mean length of inpatient stay for patients with at least one hospitalization was 9.0% shorter (95% CI: 4.05 to 20.48, P = 0.014) in the peri-COVID-19 group compared to the pre-COVID-19 group. This is due to policy changes that occurred at the national level, which impacted hospitals in striving for early discharge to keep bed occupancy low, in preparation for any unforeseen influx of COVID-19 patients. Similarly, studies found shorter mean length of inpatient stay after the introduction of telemedicine during the COVID-19 pandemic [16].

Patients who have recently been initiated on HD, incur increased risks of vascular access-related complications and failure [17]. It is important for dialysis care teams to detect any signs of impending complications early. During the peri-COVID-19 period, a higher proportion of hospitalizations were due to vascular access-related reasons as compared to the pre-COVID-19 period (OR 1.59, 95% CI: 1.10 to 2.29, P = 0.014). Given the challenges of teleconsultations, it is possible that dialysis center staff may have been more cautious, resulting in more patients being referred to hospitals for vascular access-related issues and evaluation [7].

Mortality did not differ between the peri-COVID-19 group and the pre-COVID-19 group (OR 1.40, 95% CI: 0.58 to 3.40, P = 0.452), suggesting that infection control measures did not have any significant negative impact on HD patient care. This is in accordance with other studies from Singapore, which have seen shown equivalent results [16].

Patient management

Dialysis Adequacy and Waste Management

There was no difference in Kt/V values between the peri-COVID-19 and pre-COVID-19 groups (P = 0.886 and 0.319, respectively), as detailed above (Table 4). This was primarily due to heightened awareness by the dialysis center staff during these enhanced measures, to carefully ensure that the frequency and duration of every patient’s HD treatment was maintained.

Bone and Mineral Disease

There was no statistically significant difference for PO4, corrected Ca, and iPTH between the peri-COVID-19 and pre-COVID-19 groups (P = 0.891, P = 0.966, and P = 0.396, respectively), as detailed in the results section (Table 4). However, ALP levels in the peri-COVID-19 group were 7.3% lower (95% CI: 1.17 to 13.02) than those in the pre-COVID-19 group (P = 0.020). We postulate that the improvement of ALP levels in the peri-COVID-19 group is due to changes in diet brought on by nationwide pandemic measures, which limited meals being bought and consumed from commercial outlets.

Anemia and Nutrition

There was no difference in hemoglobin levels between patients in the peri-COVID-19 group and the pre-COVID-19 group (P = 0.372), indicating that anemia was appropriately managed despite the infection control measures. Although not a main outcome of interest, the proportion of patients on iron supplementation at the end of the study period was lower in the peri-COVID-19 group compared to the pre-COVID-19 group (Table 2). This may be due to more nutritious diet being consumed during the COVID-19 pandemic, due to decreased access to commercially prepared food, fast food outlets, and snack vendors.

Patients in the peri-COVID-19 group had albumin levels 2.13% (95% CI: 0.88 to 3.39) higher than patients in the pre-COVID-19 group (P ≤ 0.001). The increase in albumin levels indicates that patients in the peri-COVID-19 group had an improved nutritional status due to better dietary intake. This observation lends support to our postulation about the reduced need for iron supplementation in the peri-COVID-19 group and the access to food factors that impacted such a change.

Aside from infection control measures implemented at dialysis centers, HD patients in Singapore were also affected by nationwide social isolation directives (lockdowns) during the DORSCON orange and yellow period. Dining outside the homes was not allowed or severely restricted and most workplaces switched to a work-from-home model [4]. We postulate that the reduced need for iron supplementation and improvement in albumin and ALP levels is due to better nutrition as an unintended result of these lock-down measures. Foods prepared outside the home are lower in beneficial micronutrients, lower in fiber, and have higher energy contributions from fat, processed meats, and processed sugars, as compared to foods prepared at home [18,19]. Lockdowns worldwide have similarly caused a variety of dietary changes in their respective local populations. Some studies reported an increase in home cooking and fresh produce consumption, whilst others reported an increase in the consumption of comfort foods, alcohol, and snacks [20]. With appropriate nutrition advice from dieticians at dialysis centers, the restrictions on dining out may have pushed HD patients to improve their diet. The commuting time saved due to work-from-home policies may have also provided more time for home food preparation. Our study did not collect diet-related data and is unable to confirm this postulation. However, in a qualitative survey on HD patients and their families which was conducted separately from this study, our colleagues found that there was improved home care and support for HD patients due to family members being on flexible work arrangements during the COVID-19 pandemic [21]. This allowed them to spend more time with the HD patient in their family at home, accompany them to dialysis centers more often and increase the consumption of home-cooked food rather than foods procured from commercial outlets. The infection control measures did not delay the intake of new patients being admitted to the dialysis centers or affected staffing in the short term, which was taken into account to assess for any bias introduced by such factors.

Strengths and limitations

This study’s main strength is the assessment of objective biomarkers or laboratory data to assess the outcomes. The main limitation is the use of a historical control group since no other appropriate control group was possible to be included during the unexpected period of the COVID-19 pandemic. Our study included only patients who had recently enrolled in the HD program at the dialysis centers and findings may not be generalizable to the overall HD patient population. Furthermore, our data is unable to track whether these patients had received chronic dialysis at other facilities prior to enrolment at the study dialysis centers. Lastly, due to the retrospective audit of patient records, some data points may be missing, which we are unable to remedy. Our study period is short and may not fully capture the long-term impacts of changes in hospitalization and improvements in dialysis outcomes. A longer study would have led to several confounders due to the rapidly changing regulations and measures in Singapore during the COVID-19 pandemic.

## Conclusions

The COVID-19 pandemic posed unique challenges to the care of hemodialysis patients in community-based dialysis centers in Singapore and worldwide. Various measures were implemented at the dialysis centers to mitigate some of the disruptive effects of nationwide healthcare directives and service disruptions. Our study showed that dialysis adequacy measures, bone mineral disease management, and anemia outcomes were unaffected by the implementation of strict infection control measures during the COVID-19 pandemic for hemodialysis patients undergoing their treatments at such centers. There was no evidence of adverse trends in hospitalizations or mortality. Furthermore, the nationwide social isolation measures led to restrictions on dining outside the homes, which combined with dietician guidance may have led to improvement in the patient's albumin and alkaline phosphatase levels. The findings from this study may help to inform future policymakers and healthcare authorities to formulate infection control measures, keeping in mind the safety and treatment outcomes of vulnerable populations, such as hemodialysis patients.
